# Policies and opportunities for physical activity engagement in Austrian schools: a census survey

**DOI:** 10.7717/peerj.17794

**Published:** 2024-08-13

**Authors:** Alexandra Unger, Andrea Schwarzenlander, Jan Wilke

**Affiliations:** 1 Department of Sport Science, University of Klagenfurt, Klagenfurt, Carinthia, Austria; 2 Department of Secondary Level, University of Teacher Education, Klagenfurt, Carinthia, Austria

**Keywords:** Physical activity, PA facilities, PA school policy, Movement friendliness, School environment

## Abstract

**Background:**

Compelling evidence suggests that schools’ infrastructure and policy represent potential predictors of health and physical activity (PA) in children and adolescents. However, the translation of these findings into practice has not been examined. This study investigated the activity friendliness of Austrian schools.

**Methods:**

Using a census sampling method, principals of Austrian schools (*n* = 342) were invited to participate in an online survey assessing 1) the availability of PA facilities (*e.g*. playgrounds, sports courts, swimming halls) and 2) applied PA policies (*e.g*. cooperation with sports clubs and involvement in PA projects).

**Results:**

A total of 130 principals answered the survey which corresponds to a minimal response rate of 38%. While most schools (87.4%, *n* = 111) had a gymnastic hall, only one third (37%, *n* = 47) had access to a swimming hall. On average, the schools had 4.2 ± 2 PA facilities with significant variation between school types (*i.e*., high schools: 5 ± 2 *vs*. primary schools: 3.5 ± 2, *p* < 0.05). The most common facilities were meadow areas (89%, *n* = 113), sports fields (71.7%, *n* = 91), and playgrounds (64.6%, *n* = 82). Almost half of the schools were part of a PA project (*e.g*. “active break”, 45%, *n* = 56) and offered extracurricular PA programs and courses (54%, *n* = 67), but only one in five (22%, *n* = 27) regularly conducted fitness tests. PA policies varied between school types (*p* < 0.05) but almost all principals (94%, *n* = 117) would welcome a stronger promotion of activity friendliness at their school.

**Conclusion:**

Schools are mostly activity-friendly regarding infrastructure although the limited possibilities for swimming lessons seem worrisome. PA promotion through projects, extracurricular PA offerings and fitness tests may be expanded.

## Introduction

Regular physical activity (PA) during childhood and adolescence has substantial benefits for physical, mental, and social health. For instance, leisure-time PA at age 9 to 15 years is associated with carotid artery elasticity during adulthood (Pälve et al., 2014) and being active as a child reduces depression risk in later life (Jacka et al., 2011). Sufficient engagement in PA, furthermore, improves sleep quality, bone structure, brain development and academic performance (Janssen & Leblanc, 2010; Poitras et al., 2016; Singh et al., 2019). Against this background, the World Health Organization (WHO) recommends a minimum of 60 min of moderate to vigorous PA per day to maintain or improve health in children and adolescents aged 5–17 years (Bull et al., 2020).

There is evidence that beneficial behavioral habits developed in childhood, *e.g*., an active lifestyle, tend to be sustained during adulthood (Fernandez-Jimenez et al., 2018). However, majority of the adolescents do not meet the PA recommendations (Guthold et al., 2020; van Sluijs et al., 2021). In Austria, according to the Health Behaviour in School-aged Children study from 2022, the WHO recommendation of at least 1 h moderate to vigorous PA is achieved on 3.8 days (girls) and 4.5 days (boys) per week only (Felder-Puig, Teutsch & Winkler, 2023).

Physical inactivity and health may not only be influenced by intrapersonal (*e.g*., age, sex, well-being, personal beliefs) but also by environmental factors (Sallis et al., 2006). Besides social support or an individuals’ economic situation, it has been argued that public infrastructure and policy play a significant role for the engagement in PA (Sallis et al., 2006). As a consequence, the spaces, facilities and opportunities provided by cities and communities could substantially influence PA levels (Kelso et al., 2021; Tcymbal et al., 2020). Insights into the locations where PA occurs, thus have the potential to support active living. Kelso et al. (2021) demonstrated the importance of an activity-friendly design of recreational locations such as parks, playgrounds and other green areas. This, arguably, also applies to schools whose activity friendliness could be affected by school policy (*e.g*. number of physical education lessons and breaks) and infrastructure (*e.g*. availability of a playground or football court) (de Rezende et al., 2015; Ferrari et al., 2021; Kelso et al., 2021; Tcymbal et al., 2020).

According to Tassitano et al. (2020), about 50% of children’s daily PA are accumulated during schooltime. A review evaluating international data found daily moderate to vigorous PA at school to range between 0 and 38 min per day with a higher amount achieved outdoors than indoors (Dessing et al., 2013; Kelso et al., 2021). In Austria, children and adolescents engage in two to four mandatory PE lessons per week, depending on the type of school and the age of the students which means that the PA recommendations cannot be fulfilled through the regular curricula. Interestingly, in Germany, the overall time spent in extracurricular sports increased from 6 to 17 min per week over the last years (Schmidt et al., 2020), but it is unclear if this is also the case in the neighboring country of Austria. There is evidence that a higher amount of PA can be seen on school days than on days without school visits (Guinhouya et al., 2009; Klinker et al., 2014; Schmidt et al., 2020). Activity promotion at school, therefore, is of paramount importance (Guinhouya et al., 2009; Hills, Dengel & Lubans, 2015).

As indicated above, a growing body of research demonstrates that engaging in PA during school time is not merely an individual’s choice but substantially influenced by the school environment (McGrath, Hopkins & Hinckson, 2015; Zhou & Wang, 2019). In fact, Zhou & Wang (2019) and de Rezende et al. (2015) found available outdoor facilities such as soccer fields or playgrounds to be associated with motor skill proficiency and PA levels in secondary schools. Tonge, Jones & Okely (2016) focused on younger children, reporting larger outdoor environments to improve PA and reduce sedentary behavior. de Rezende et al. (2015) identified an association of the number of PA facilities (*e.g*. sports courts, running tracks, swimming pools) and PA in adolescents: while at least two facilities were suitable to increase total PA, at least four were required to enhance leisure-time PA.

In addition to the availability of PA facilities, school policy appears to represent an essential factor in activity promotion. A variety of studies underscored the importance of intra- and extracurricular activities during school time for promoting a healthy lifestyle (Bonell et al., 2013a, 2013b; de Rezende et al., 2015; Morton et al., 2016; Tassitano et al., 2020). Others suggested policy measures for schools to increase PA, including involvement in projects such as active school or active commuting (Klos et al., 2023; Messing et al., 2019; Norris et al., 2020). Despite the solid evidence underpinning the value of an activity-friendly school environment, infrastructure and policy (de Rezende et al., 2015; Messing et al., 2019; Norris et al., 2020) high quality research is urgently needed. With regard to Austria, there is a paucity of data on PA opportunities provided at and during school. Our investigation therefore aimed to analyze the availability and accessibility of PA facilities (*e.g*. sports fields, playgrounds, athletics facilities) and the PA policy as reported by schools in a state of Austria.

## Materials and Methods

### Ethics and study design

The Carinthian Schools’ Physical Education Report (CSPER) comprised a structured survey administered to all principals of schools in the Austrian state Carinthia. The study, which followed the guidelines for Good Practice in the Conduct and Reporting of Survey Research (Kelley et al., 2003), was reviewed and approved by the Ethics Council of the University of Klagenfurt (ref.no. 2022-075). All participants provided implied and informed consent in the online survey.

### Sample

To evaluate the availability of infrastructural PA opportunities and the schools’ policies with regard to PA, we applied a census sampling method, inviting all 342 principals heading primary schools, middle schools, high schools and vocational schools in Carinthia. Schooling in Austria is mandatory for 9 years, from age 6 to 15 years (first to ninth grade). The first 4 years of education are completed in primary school at age 6 to 10 years. From age 10, children may attend a middle school or a high school, comprising four educational levels. After this period (4 years), children may a) stay in high school until 18 years with the aim of achieving the general school-leaving examination or b) choose a higher vocational school with a higher qualification in profession or occupation and school-leaving examination at the age of 19.

In collaboration with the local school authority, the principals were emailed with a link to the online survey on 16th January 2023. To increase response rate, a reminder email was sent 2 weeks later, on 3rd February 2023 (Sammut, Griscti & Norman, 2021).

### Instrument

The CSPER questionnaire was generated using a multi-stage expert consensus process. Initially, all authors gathered ideas and possible survey items which were then grouped, edited and reduced to produce a first version of the instrument. The resulting draft was then sent to the state’s department of education for further external feedback. After revising and adapting the questionnaire, it was passed on to three members of the target population (one principal of a primary, middle, and high school each) for face validation. All reported high comprehensibility and clarity but provided some minor suggestions for improvement.

The final instrument comprised a combination of single- and multiple-choice questions relating to the activity friendliness of schools. The first part addressed basic sociodemographic data such as the school type, environment of the school (urban/rural) or the number of students. The second part of the CSPER questionnaire had two main focuses. The *infrastructure* section assessed the availability of a school yard, a gymnastic and swimming hall as well as the availability of other facilities for PA promotion (*e.g*. meadow areas, sports fields, playgrounds, ping pong tables, basketball hoops, beach volleyball courts, climbing walls, skate parks and gyms). The PA *policy* section asked questions on break duration, the educational level of PE teachers, cancellation of and student participation in PE classes. In addition, we assessed the schools’ involvement in PA promotion projects, extracurricular PA programs, sports events (*e.g*., school championships, sports badges), cooperations with local sports clubs, and performance of fitness tests. The questionnaire was concluded by Likert-scaled questions used to capture satisfaction with school infrastructure (very dissatisfied = 1, rather dissatisfied = 2, rather satisfied = 3, very satisfied = 4) and the school’s activity friendliness (strongly disagree = 1, tend to disagree = 2, agree somewhat = 3, agree completely = 4).

### Statistical analysis

The normal distribution of data was tested by means of the Shapiro–Wilk test. Descriptive statistics were then applied to analyze the obtained data (means/medians) as appropriate. Interval/quasi-interval scaled data (*e.g*. number of students, duration of the longest break) and dichotomous data (*e.g*., PA facilities, additional sport activities) were presented as absolute (*n*) and relative (%) values, respectively.

In addition to descriptive analyses, we examined differences between school types for the mean number of PA facilities (ANOVA), availability of individual PA facilities, use of specialized PE teachers, participation in PE lessons, cancellation of PE classes, PA policies, rated activity friendliness (all chi squared test), school environment (urban/rural, Mann-Whitney-U test) and extracurricular PA programs (yes/no, Mann-Whitney-U-test). Pearson and Spearmen correlations were performed to reveal potential associations between the number of PA facilities and the number of students, break length, as well as satisfaction with PA facilities and school’s activity friendliness. The significance level was set to α = 0.05. Data analyses were performed using JAMOVI, version 2.3 (https://www.jamovi.org).

## Results

### Sample characteristics

A total of 130 principals completed the survey which corresponds to a minimal response rate of 38%. The majority of the respondents (54%, *n* = 71) were employed at primary schools, while the remainder directed middle schools (21.5%, *n* = 28), high schools (10.8%, *n* = 14) and vocational high schools (13.1%, *n* = 17). About one third (37.7%, *n* = 49) of the schools were located in urban areas; 62.3% (*n* = 81) were from rural areas. School size ranged from 11 (primary school) to 1,294 (high school) students (median: 132).

### Infrastructure and PA facilities

School yards (at least 4 m² per student) were reported to be available by 75.6% (*n* = 96) of the principals. Almost all schools (87.4%, *n* = 111) had a gymnastics hall (median size: 235 m^2^), but only one third (37%, *n* = 47) had access to a swimming hall.

The mean number of PA facilities per school (excluding school yard and gymnastic/swimming hall) was 4.2 ± 2 (min: 0, max: 9). Significant differences were observed between school types (*p* < 0.05) as primary schools had less facilities (3.5 ± 1.7) than others (high schools: 5.1 ± 2, middle schools: 4.9 ± 1.9, vocational high schools: 4.5 ± 2.3).

The most frequent PA facilities were meadow areas, sports fields and playgrounds. In contrast, gyms and skate parks were least prevalent (Table 1). Similar to the total number, the individually available facilities differed between school types (Table 2): Meadow areas were most prevalent in primary and middle schools (*p* < 0.05); the same applied to playgrounds (*p* < 0.05). In contrast, beach volleyball courts were more often found in high schools and vocational high schools (*p* < 0.05). Athletics facilities were frequent in all types of schools, except for primary schools (*p* < 0.05, Table 1).

**Table 1:** PA facilities stratified by school type.

	Primary school (*n* = 68)		Middle school *n* = (28)		High school (*n* = 14)		Voc.high school (*n* = 17)		Total sample (*n* = 127)		Chi squared test	
Facility	*n*	%	*n*	%	*n*	%	*n*	%	*n*	%	χ2	*p*
Meadow area	64	94.1	26	92.9	12	85.7	11	64.7	113	89	12.6	**0.006**
Gymnastic hall	64	94.1	25	89.2	8	57.1	14	82.3	111	87.4	21.7	**0.01**
School yard	59	86.7	20	71.4	10	71.4	7	41.2	96	75.6	33.2	**<0.001**
Sports field	44	64.7	23	82.1	11	78.6	13	76.5	91	71.7	3.66	0.3
Playground	56	82.4	20	71.4	4	28.6	2	11.8	82	64.6	38.6	**<0.001**
Table tennis	21	30.9	20	71.4	8	57.1	7	41.2	56	44.1	14.3	**0.002**
Athletics facility	14	20.6	18	64.3	12	85.7	10	58.8	54	42.5	31.3	**<0.001**
Basketball hoops	22	32.4	11	39.3	8	57.1	10	58.8	51	40.2	5.88	0.11
Swimming hall	26	38.2	10	35.7	5	35.7	6	35.2	47	37	0.09	0.99
Beachvolleyball court	7	10.3	10	35.7	8	57.1	12	70.6	37	29.1	31.7	**<0.001**
Climbing wall	16	23.5	7	25	5	35.7	6	35.3	34	26.8	1.61	0.66
Skate park	7	10.3	3	10.7	1	7.14	1	5.88	12	9.4	0.44	0.93
Gym	0	0	1	0.03	2	14	6	35.2	9	7.1	27.4	**<0.001**

**Note:**

The table lists absolute (*n*) and relative (%) frequencies per column. PA = physical activity, Voc. = vocational, significant *p* values are marked bold.

**Table 2:** Relationship between the number of PA facilities and other variables.

Variable	Effect size		*p*
	Difference	Correlation	
School type	f = 5.38		**0.004**
Number of students		r = 0.22	**>0.001**
School environment	u = 0.05		0.64
Break length		r = 0.01	0.87
Participation in PE lessons		rho = −0.19	**0.02**
Extracurricular PA programs	u = 0.36		**>0.001**
Satisfaction with PA facilities		rho = 0.04	0.65
Satisfaction with activity friendliness		rho = 0.007	0.94

**Notes:**

The table shows either associations of the number of PA facilities with other variables or differences in the number of PA facilities as a function of other variables. Significant *p* values are marked in bold. PA = physical activity, PE = physical education.

No difference in the number of PA facilities was found for the school environment (urban *vs*. rural, *p* > 0.05). However, correlation analyses revealed a significant positive association between the total number of PA facilities and the number of students (*p* < 0.001, r = 0.22, Table 2).

Two thirds of the principals (66.1%) were satisfied with the PA facilities of their school but satisfaction did not correlate with the number of facilities (*p* = 0.65). Likewise, no association was found between the number of PA facilities and satisfaction with the general activity friendliness of schools (*p* > 0.05, Table 2).

### PA policy

The median duration of the longest break was 15 min (interquartile range/IQR: 5). High schools reported the lowest duration (10 min, IQR = 5) while vocational high schools had the longest breaks (30 min, IQR = 25). No association was found between the number of PA facilities and break length (*p* > 0.05).

With regard to the PE teachers’ educational level, three quarters of the schools reported appointment of qualified teachers only, while almost one quarter had classes taught by both partly-qualified and non-qualified (no academic degree in PE) personnel (Table 3). Middle schools demonstrated the lowest percentages of PE classes taught by qualified PE teachers (42.8%), while high schools had the highest percentages (100%, *p* < 0.05).

**Table 3:** Physical education classes stratified by school type.

	Primary school (*n* = 70)		Middle school (*n* = 28)		High school (*n* = 14)		Voc. high school (*n* = 17)		Total sample (*n* = 129)		Chi squared test	
Policy	*n*	%	*n*	%	*n*	%	*n*	%	*n*	%	χ2	*p*
**Teachers’ education**												
Qualified teachers	54	77.1	12	42.8	14	100	16	94.1	96	73.8	27.3	**<0.001**
Partly-qualified teachers	17	24.2	15	53.5	0	0	0	0	32	24.6		
Non-qualified teachers	0	0	1	3.5	0	0	1	5.8	2	1.5		
**Cancellation of PE classes**												
<20%	68	97.1	26	92.8	13	92.8	13	76.4	120	93	23.4	**0.024**
>20%	1	1	1	3.5	0	0	3	17.6	5	4		
I don't know	1	1	1	3.5	1	7.1	1	5.8	4	3.2		
**Participation in PE lessons**												
>80%	65	92.8	22	78.5	12	85.7	9	52.9	108	83.7	37.1	**0.001**
<80%	4	5.71	6	21.42	1	7.14	8	47.05	19	14.9		
I don’t know	1	1.4	0	0	1	7.1	0	0	2	1.6		

**Notes:**

The table lists absolute (*n*) and relative (%) frequencies. Voc. = vocational, PE = physical education, significant *p* values are marked in bold.

In most schools, PE classes were cancelled less than 20% of the time due to other events or timetable shifts, but cancellations were highest in vocational high schools (*p* < 0.05). More than four in five schools had at least 80% of students participating actively in PE lessons. Vocational high schools showed the lowest participation rate (52.9%, *p* < 0.05). Lower participation rates were associated with higher PA facility counts (*p* = 0.02, rho = −0.19).

With regard to PA promotion strategies, about half of the schools (45.2 %) reported being part of a PA project and likewise, more than half (54%) of the respondents stated offering extracurricular PA programs and courses (Table 4). The majority of the schools confirmed both participation in sports events (66.9%) and cooperation with a local sport club (55.6%). One fifth of the schools reported regular performance of fitness tests.

**Table 4:** PA policies and rated activity friendliness stratified by school type.

	Primary school (*n* = 67)		Middle school (*n* = 28)		High school (*n* = 13)		Voc. high school (*n* = 16)		Total sample (*n* = 124)		Chi squared test	
Policy	*n*	%	*n*	%	*n*	%	*n*	%	*n*	%	χ2	*p*
Fitness tests	13	19.4	8	28.6	4	30.8	2	12.5	27	21.8	2.41	0.49
Participation in sports events	38	56.7	18	64.3	13	100	14	87.5	83	66.9	12.7	**0.005**
Cooperation with sports clubs	48	71.6	11	39.3	6	46.2	4	25	69	55.6	16.5	**<0.001**
PA promotion projects	46	68.7	6	21.4	2	15.4	2	12.5	56	45.2	32.8	**<0.001**
Extracurricular PA programs	23	34.3	23	82.1	13	100	8	50	67	54	30.5	**<0.001**
Wish for professional support in PA promotion	61	91	28	100	13	100	15	93.8	117	94.4	3.84	0.28
Rated activity friendliness*												
Agree completely	43	64.2	6	21.4	4	30.8	0	0	53	42.7	40.2	**<0.001**
Agree somewhat	22	32.8	17	60.7	7	53.8	10	62.5	56	45.2		
Tend to disagree	2	2.99	5	17.9	2	15.4	5	31.3	14	11.3		
Strongly disagree	0	0	0	0	0	0	1	6.25	1	0.8		

**Notes:**

The table lists absolute (*n*) and relative (%) frequencies. Voc. = vocational, PE = physical education.

* Answer to the question “How much do you agree with the statement that your school is activity friendly?”. Significant *p*-values are marked in bold.

PA promotion strategies were significantly different between school types except for the performance of fitness tests (*p* = 0.49). While primary schools were most frequently part of a PA promotion project and collaborated most frequently with local sports clubs (*p* < 0.05), high schools had the highest participation in sports events and the highest number of extracurricular PA programs (*p* < 0.05, Table 4). Schools offering more extracurricular PA opportunities had higher PA facilities (u = 0.36, *p* < 0.001).

Almost all principals (94.4%) would welcome professional support for PA promotion at their school and this wish was not different between school types (*p* = 0.28). More than 80% of the principals agreed their schools were activity-friendly (Table 4) and complete agreement with this statement was highest in primary schools (*p* < 0.05).

## Discussion

Schools, if designed to be activity-friendly, represent pivotal elements of PA promotion for youth (Bonell et al., 2013a; de Rezende et al., 2015; Morton et al., 2016; Tassitano et al., 2020). A variety of measures can support children and adolescents in achieving the required PA levels according to the WHO recommendation and these include the provision of outdoor facilities (*e.g*., playgrounds or sports fields), offering of extracurricular courses, or the involvement in projects such as active transport or active school. Of note, previous literature (Carrasco-Uribarren et al., 2023; Hu et al., 2021; Masini et al., 2020; Morton et al., 2016) had predominantly focused on the effects of specific PA interventions in individual schools, but little research has investigated the availability and actual use of PA promotion strategies from a more population-based perspective (Amornsriwatanakul et al., 2021; de Rezende et al., 2015; Morton et al., 2016).

To the best of our knowledge, this is the first assessment of schools’ activity friendliness that aimed to gauge the current situation of PA facilities and school policy in four different types of schools in Austria. Our main finding, based on an analysis of responses from primary, middle, high and vocational high schools, is that the educational institutions in Carinthia seem mostly well-equipped with regard to infrastructural aspects as the mean number of PA facilities was 4.2 ± 2 across the investigated sample. According to Ferrari et al. (2021) the level of PA is 3–4 times higher in schools with more facilities (≥3) than in schools with fewer facilities. With regard to the specific number, Haug et al. (2010) found out that four of eight studied infrastructural characteristics (soccer fields, areas for hopscotch/skipping rope, playground equipment and sledding hill) were significant predictors for daily PA. de Rezende et al. (2015) reported four or more facilities to increase leisure-time PA. As our study showed a mean number of four activity facilities, the average school in Carinthia appears to provide sufficient infrastructural PA promotion. Of note, the number of PA facilities seems to be more important for promoting PA than provision of PE classes itself (Sales et al., 2023). This could be explained by the freedom of choice in doing preferred activities alone or in groups contrary to the rigid and organized structure of PE classes (de Rezende et al., 2015; Haug et al., 2010). Interestingly, our results showed a negative correlation between the participation rate in PE lessons and the number of PA facilities. Although the size of the effect was small, this finding is in contrast to previous studies indicating that students spend more time in PE classes with better PA infrastructure (Coledam et al., 2014; de Rezende et al., 2015; Zhou & Wang, 2019).

Total PA facility counts were different between school types. We found the largest number in high schools while primary schools had the lowest. As mentioned, environmental characteristics are contributors to daily PA (Amornsriwatanakul et al., 2021; Ferrari et al., 2021) and primary schools are particularly important in combating the decline in PA as the decrease begins at the transition from early childhood to primary school (Carrasco-Uribarren et al., 2023; Chong et al., 2020; Steene-Johannessen et al., 2020; Weaver et al., 2021). Improving the amount, variety and condition of activity facilities in primary schools is a highly relevant strategy to increase PA levels throughout the school career (de Rezende et al., 2015; Tonge, Jones & Okely, 2016). Therefore, particularly primary school may be considered when investing into the activity friendliness of schools.

The analysis of schools’ PA policies draws a slightly different picture. Although the situation may be considered satisfactory, a significant portion of schools do not promote PA using projects (54.8%), cooperations with sports clubs (44.4%), participation in sport events (33.1%), or regular fitness tests (78.2%). Our study also identified differences in PA policy between school types. Again, high schools showed greater levels of PA promotion. These findings align with a previous study (Morton et al., 2016) which showed a more positively perceived physical environment, and a greater amount of extracurricular physical activity offerings in high schools than in primary schools. High schools, in our study, also had the highest percentage of qualified PE teachers. It is well established that children taught by PE specialists have a higher activity level resulting in a higher energy expenditure rate during PE classes (Ferrari et al., 2021; Martin et al., 2014).

The importance of break times has been outlined in the previous literature (Murray & Ramstetter, 2013; Parrish et al., 2020; Sales et al., 2023). However, Baines & Blatchford (2023) reported marked changes between 1995 and 2017 in the total time of school breaks in the United Kingdom: In primary schools, a weekly reduction of about 40 min of breaktime was seen, and in secondary schools, the decrease amounted more than 1 h. The morning break duration of 15–20 min in both primary and secondary schools is consistent with our findings (overall mean duration of 15 min). However, interestingly, we documented shorter breaks in high schools (10 min). As there is a positive association between break duration and PA levels (Lau et al., 2017), the Carinthian high schools may consider implementing higher break lengths.

Our study has practical implications, underscoring the need for appropriate infrastructure and PA promotion in schools to act as places for sustainable health education. We showed that the provision of PA infrastructure may depend on the type of school and while the number of facilities is generally high, primary schools display lower counts than other types of schools. Seeking to increase facilities in primary and middle schools could hence more effectively support children’s and public health.

However, merely providing infrastructure may not be sufficient to fully exploit the potential of PA promotion in schools. We recommend the involvement of schools in PA projects and initiatives which aim to provide children with sufficient time to engage in PA (*e.g*., longer break durations and/or extracurricular PE lessons).

### Limitations

Some methodological aspects merit discussion. A particular strength of our study is that we invited all principals of Carinthia to participate in the survey. The resulting response rate of 38%, at first glance, is at best satisfactory (Wu, Zhao & Fils-Aime, 2022). Yet, it needs to be considered that it represents a conservative estimate. First, although the questionnaire was distributed by the education directorate, some dead email addresses may have been included in the sending list. Second and more importantly, not all invited participants may have seen, opened and read the emails, particularly because we did not use a reading confirmation. The distribution of school types and school sizes in our sample corresponded strongly to the distribution of all schools in Carinthia. However, although our participants seem to reflect the true population and despite the the likely higher true response rate, sample representativeness of the entire Carinthia principal population cannot be safely assumed. Nor can this sample be suggested to represent principals in other states in Austria or in other countries.

Another issue relates to the source of the information. We decided to approach school principals as the leaders who would have the most “whole school” perspective. PE teachers may have more granular-level knowledge about what happens in their classes and elsewhere with PA but this could also have skewed their responses about the school facilities and policies (positively or negatively). We also acknowledge that in some cases, principals may not have had sufficient insight into the micro-level PA promotion (*e.g*. in the PE classes). Therefore, future studies may conduct similar surveys with other stakeholders such as teachers, students, and parents.

Finally, it may have been intriguing to assess more background variables such as the socioeconomic background of the community in which schools are located or the social status of the families. As it has been shown that social inequality is related to PA behavior, it is critical to know which social mechanisms enable or prevent the participation of children and adolescents in PA (Andersen & Bakken, 2019; Rittsteiger et al., 2021; Wijtzes et al., 2014).

## Conclusions

Schools in Carinthia are mostly well-equipped with regard to PA infrastructure (*i.e*. the number of PA facilities), but this does not apply to primary schools. Schools’ PA policy requires improvement as only half of the schools are offering extracurricular PA programs or PA promotion projects. Particularly, primary schools display a need for action in view of the smaller number of PA facilities and the lower use of PA promotion strategies.

## Supplemental Information

10.7717/peerj.17794/supp-1 Supplemental Information 1 Raw data.

10.7717/peerj.17794/supp-2 Supplemental Information 2 An explanation of the numerical data and variables used in the raw data. The legend provides definitions for each item of the raw data to enable a better understanding and verification of the results.

10.7717/peerj.17794/supp-3 Supplemental Information 3 Survey questions.

## Additional Information and Declarations

### Data Availability

The following information was supplied regarding data availability: The raw data from the survey are available in the Supplemental File.
