# Supplementary material for: Policies and opportunities for physical activity engagement in Austrian schools: a census survey
Source: PeerJ. 2024 Aug 13;12:e17794. doi: 10.7717/peerj.17794 (PMC11328834; doi:10.7717/peerj.17794)
Supplement: Supplemental Information 3 [file peerj-12-17794-s003.docx]

**Carinthian Schools’ Physical Education Report (CSPER)**

**General**

Please indicate your school type (one choice):

- Primary school

- Middle school

- High school)

- Vocational high school

Total number of pupils:

______

Environment of your school:

- rural

- urban

Who teaches Physical Education at your school?

-Specialised teachers

-Partly- specialised teachers

-Non- specialised teachers

Duration of the longest break (minutes)?

_________

**Physical education classes**

Which percentage of PE classes is cancelled at your school?

- 0-20%

- 20-40%

- 40-60%

- 60-80%

- 80-100%

- I do not know

What is the average participation rate in PE lessons?

- 0-20%

- 20-40%

- 40-60%

- 60-80%

- 80-100%

- I do not know

**Physical activity infrastructure**

Does your school have a gymnastic hall?

- Yes, own

- Yes, rented but for sole use

- Yes, rented for shared use (e.g. with a club or other schools)

- No

How many gymnastic halls does your school have (rented and owned)?

___________

-

How big is the largest gymnastic hall your school uses? (approx. in m²)

___________

-

Does your school have access to a swimming hall in the immediate surrounding (<5km)?

- Yes

- No

Does your school have a school yard of at least 4 m² per pupil (for 100 pupils: 400 m²)

- Yes

- Yes, but it is smaller than 4 m² per pupil

- No, no school yard available

Please indicate which facilities are available at your school outside the gymnastic hall (either owned or easily usable, multiple choice possible):

- Meadow area of at least 200 m²

- Playground (at least 1 climbing frame and a swing)

- Table tennis table(s) (independent of gym/sports field)

- Athletics facility (field with long jump facility, 100 m plug, throwing facility, etc.)

- Gym/fitness room

- Climbing wall/bouldering wall

- Beach volleyball court

- Skate/roller skating facility

- Outdoor sports field (football/basketball/handball etc.)

- Basketball hoops (independent of gym/sports field)

- Others:

How satisfied are you with PA facilities at your school? (Scale: 1 = not at all satisfied, 10 = very satisfied)

- very dissatisfied

- rather dissatisfied

- rather satisfied

- very satisfied

**Extracurricular sports activities**

Regular fitness tests are carried out at the school and the results are documented:

- Yes

- No

Sports events (e.g. school championships, Turn 10, sports badges) are held regularly (at least once annually):

- Yes

- No

Is there an official cooperation between your school and a sports club?

- Yes

- No

Is the school part of a structured physical activity project (e.g. "Active School", "Active Break ", “Active Transport”)?

- Yes

- No

Does your school offer other permanent extracurricular PA programs or courses?

- Yes

- No

**Concluding general questions**

How much do you agree with the statement that your school is activity friendly?

- strongly disagree

- tend to disagree

- Agree somewhat

- Agree completely

Would you wish to enhance the PA promotion at your school?

- Yes

- No
